# Supplementary material for: Age and structure of a model vapour-deposited glass
Source: Nat Commun. 2016 Oct 20;7:13062. doi: 10.1038/ncomms13062 (PMC5080435; doi:10.1038/ncomms13062)
Supplement: Supplementary Information — Supplementary Figures 1-32 and Supplementary Table 1 [file ncomms13062-s1.pdf]

Supplementary Figures

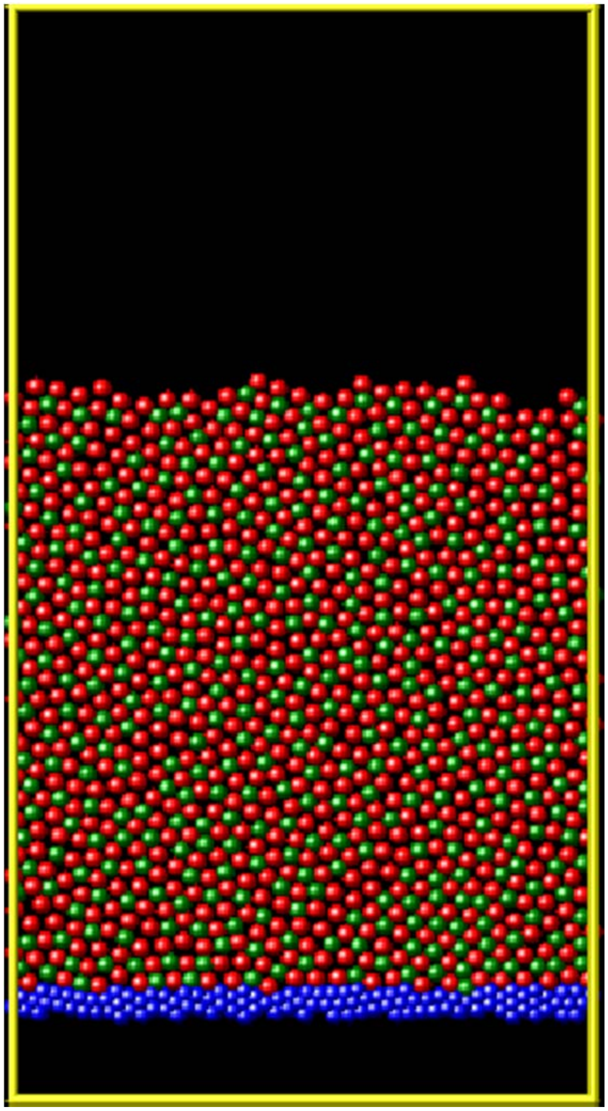

Figure 1: A sample vapor deposited configuration generated using t_{dep} = 1.4X10^4 τ_α with T_s = 0.67 T_g. This film represents one of our most stable configurations. Type A particles are shown in red while type B particles are shown in green.

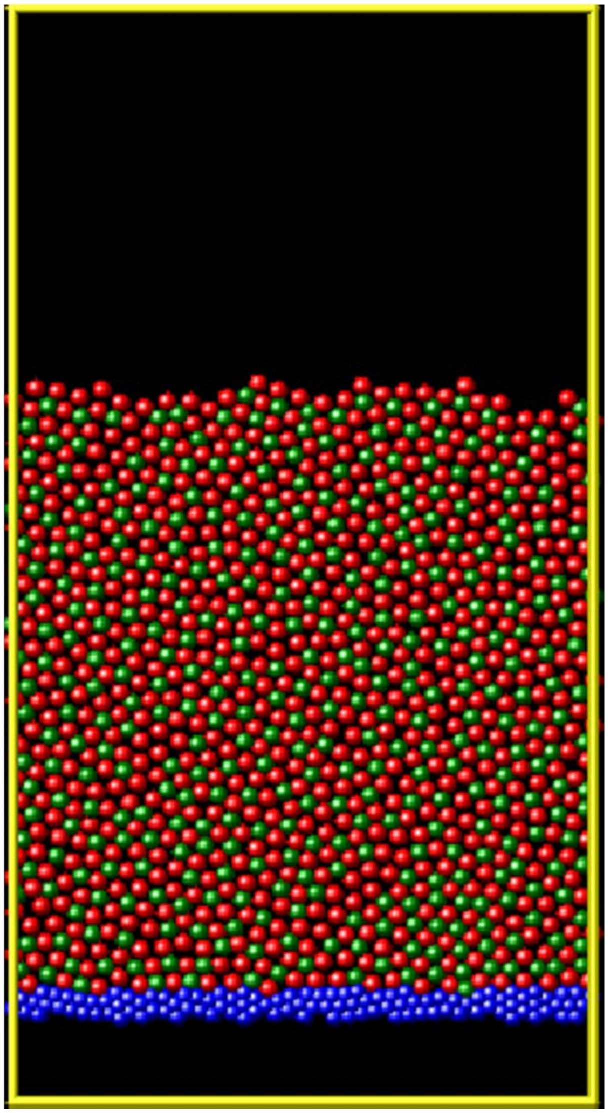

Figure 2: A sample liquid cooled configuration generated using t_{cool} = 1.4X10^{1} τ_α taken at T=0.25 T_g. This film is among our less stable configurations. Type A particles are shown in red while type B particles are shown in green.

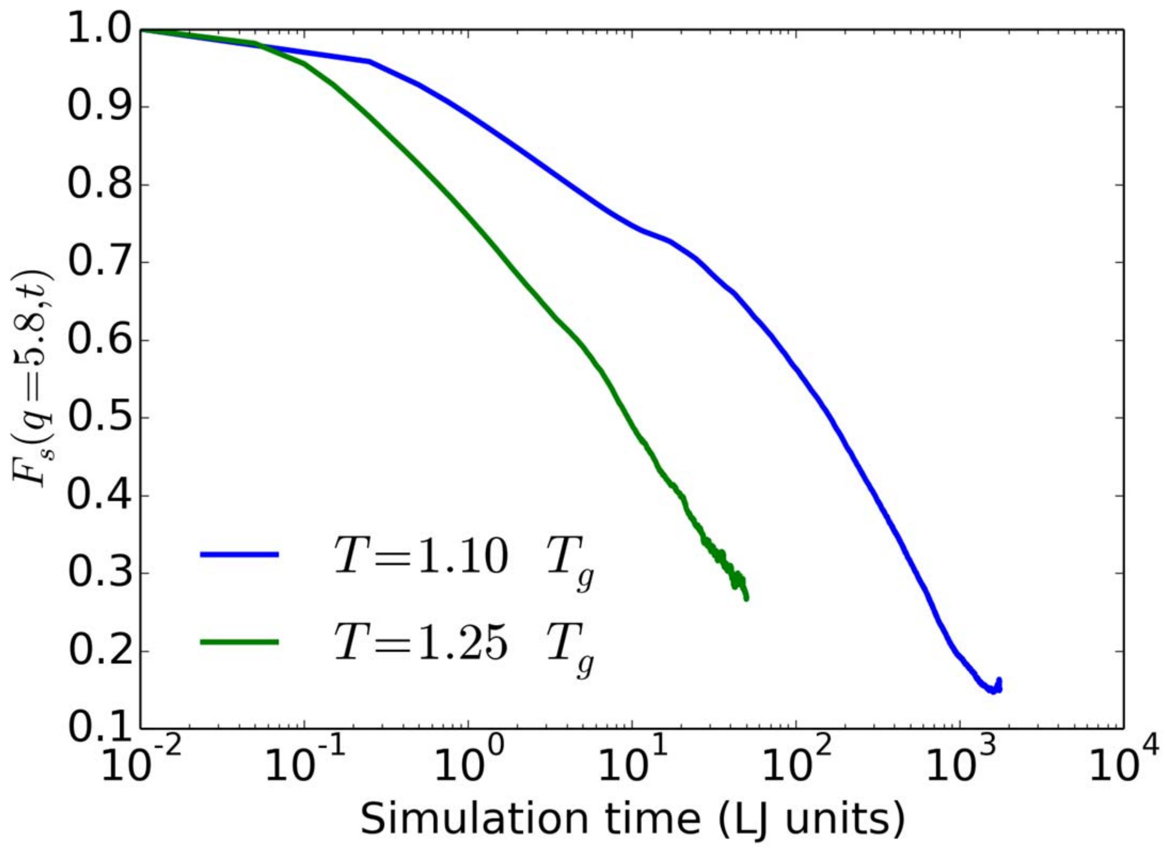

Figure 3: Self-intermediate scattering function for 2D films at T = 1.1 T_g and T=1.25 T_g calculated with q=5.8, as determined from the static structure factor. τ_α is taken to be where the value of the self-intermediate scattering function decays to 1/e. We normalize all simulation times by τ_α taken at T=1.1 T_g, 370 Lennard-Jones time units.

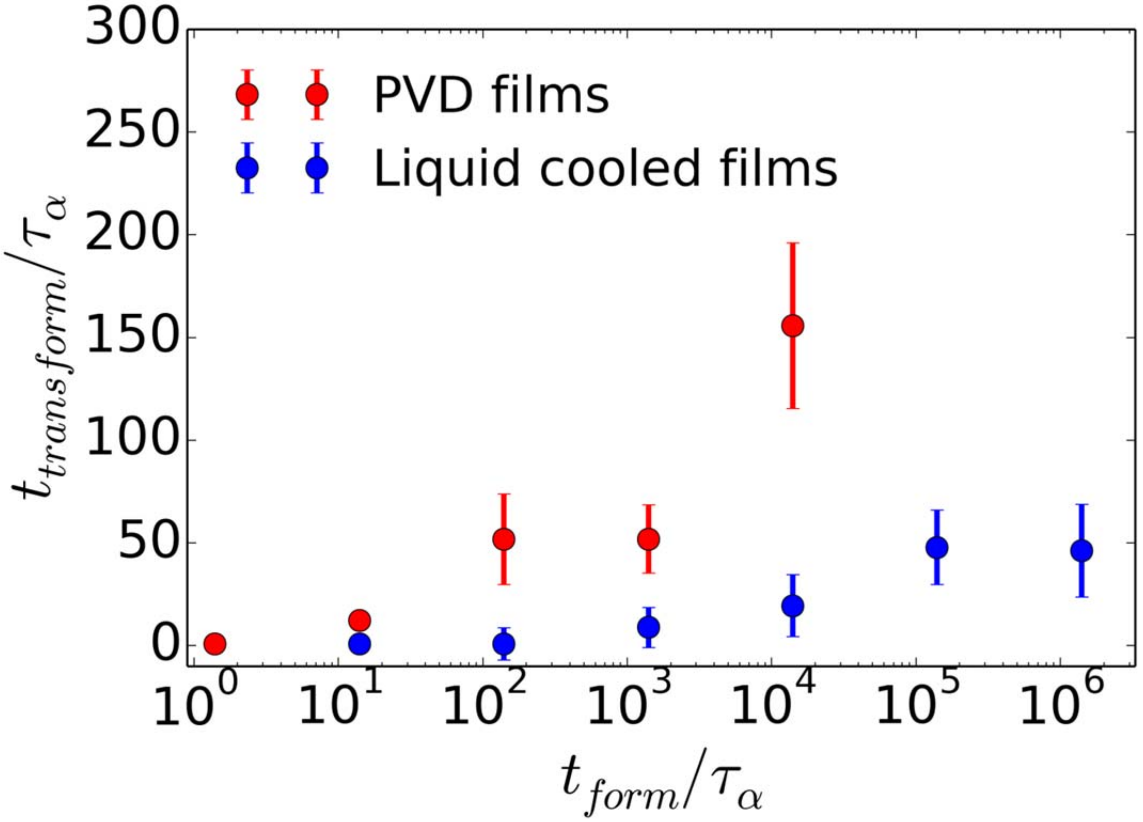

Figure 4: Transformation times of PVD and liquid-cooled films measured at T=1.1 T_g. Transformation times are normalized by τ_α measured at T=1.1 T_g. The characteristic formation time of the films, t_{form}, refers to t_{dep} for PVD films and t_{cool} for liquid-cooled films. Error bars represent standard deviations of transformation times.

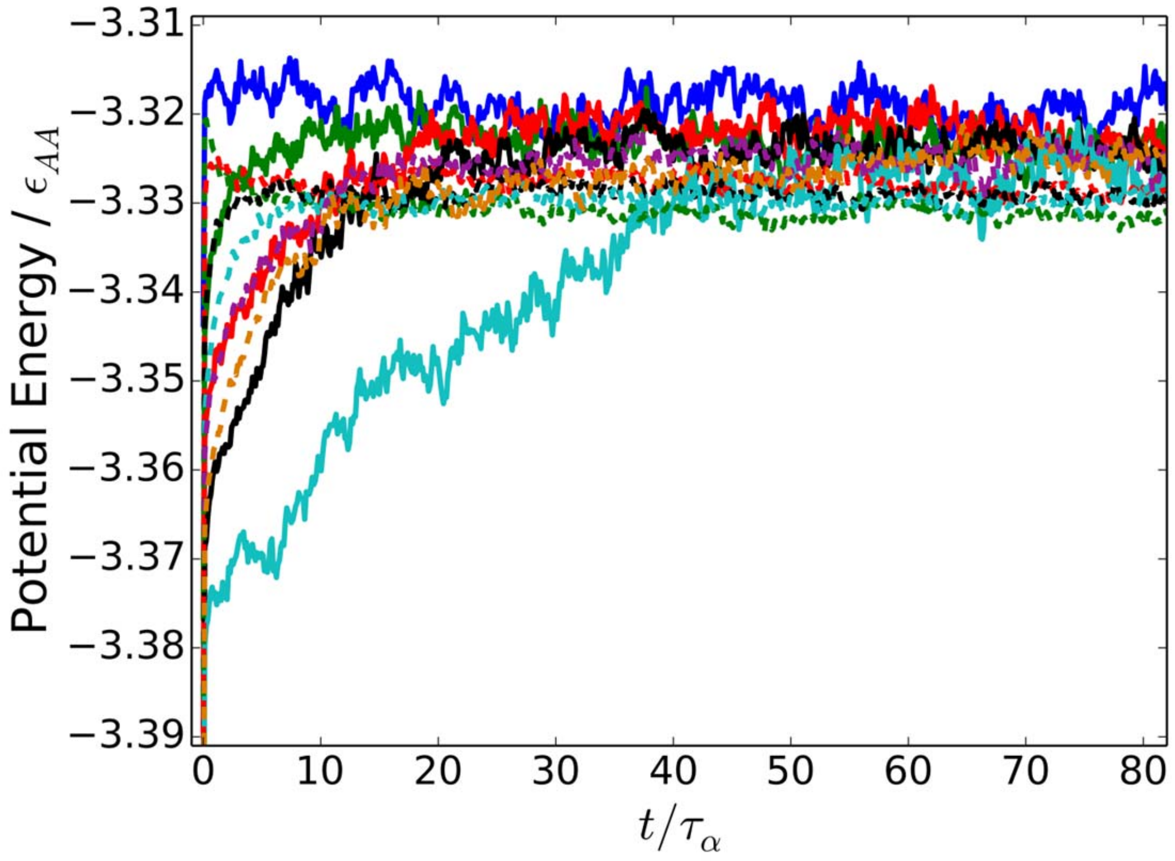

Figure 5: Potential energy vs. time for films which have been quickly heated to 1.1 T_g. Energies are used to calculate film transformation times shown in Figure 4 in Supplementary Information and referenced in the main text. Dashed lines represent liquid cooled data while solid lines represent vapor deposited data. Legend values refer to t_{cool} or t_{dep} for a given data set, in units of τ_α (calculated at T=1.10 T_g). The transformation time is defined to be when the film has moved 90% of the way from its initial energy to its equilibrium energy. This is a two stage melting process. In the first stage, which we observe here, locally ordered structures melt. In the second, film composition will re-equilibrate given the new temperature. As described in the main text, the composition of these films is not uniform, and can depend on formation method and rate. Thus energies after the first stage of melting are not all precisely equal.

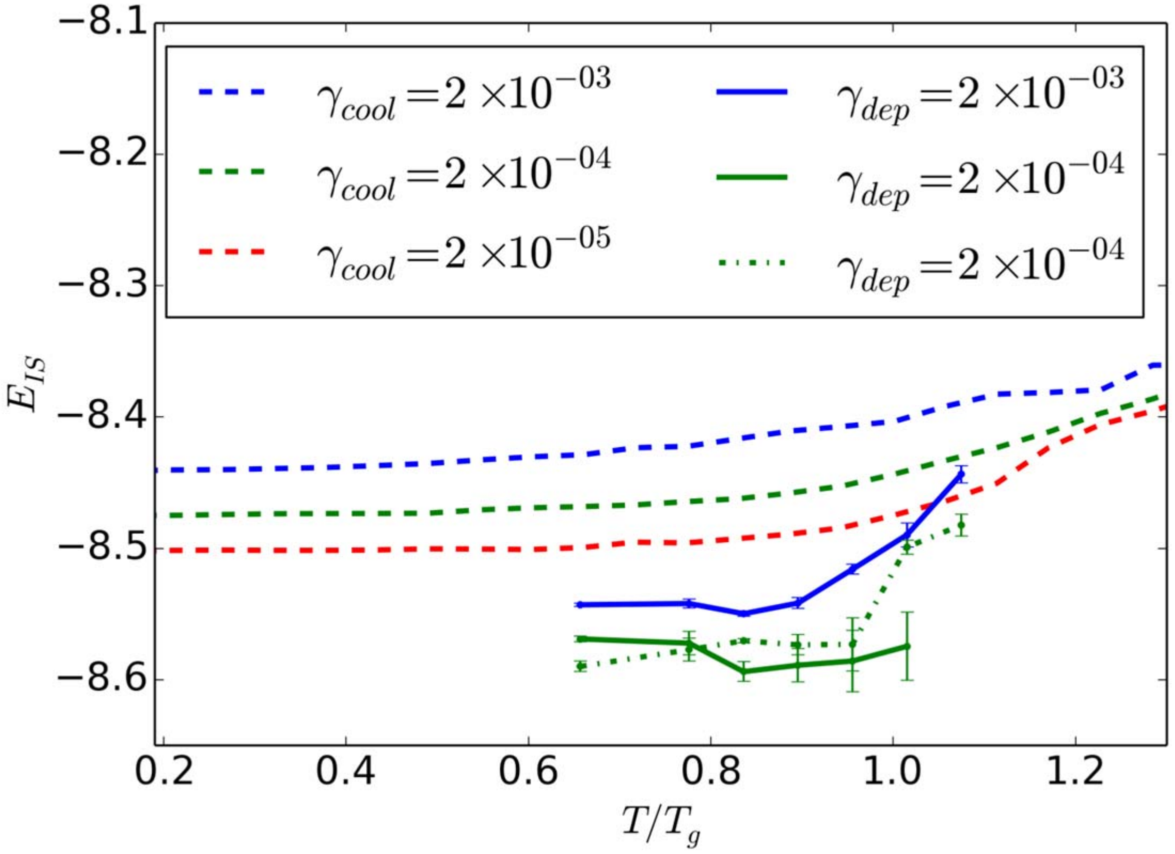

Figure 6: Inherent structural energies for liquid cooled, NVE deposited, and NVT deposited films for 3D glass films. We find that NVE deposition is at least as efficient as NVT in 3D as well. We use the standard 3D Kob-Andersen model, with χ=0.8, and deposit at several rates and substrate temperatures. Figure 24 shows that NVE energies are at least as low as in NVT Dashed lines represent liquid cooled films, solid lines represent PVD films formed with NVE deposition, which dotted lines represent PVD films formed with NVT deposition. The data shows that both NVE and NVT deposition produce highly stable films. The data also shows that NVE deposition is at least as effective as NVT, making it a preferable method as it is more physically realistic. Error bars represent standard error. The cooling or deposition rate is represented by γ, which is in Lennard-Jones units.

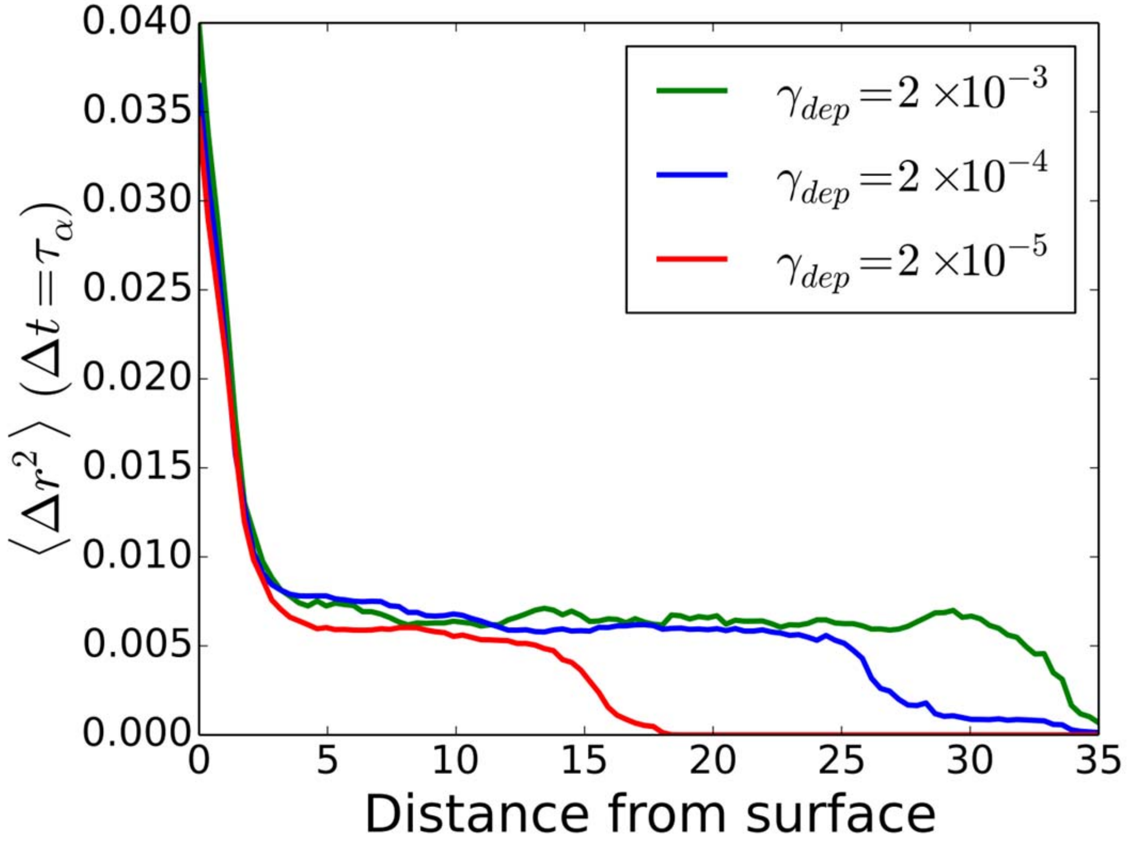

Figure 7: Mobility data for 3D PVD films formed at several deposition rates with T_s = 0.8 T_g. Films formed with slower deposition rates are more stable and show slightly lower mobilities. The deposition rate is labeled as γ_{dep}. Note that the films formed with γ_{dep} = 2.0X10^{-5} are thinner than others, thus the data does not extend as far from the surface.

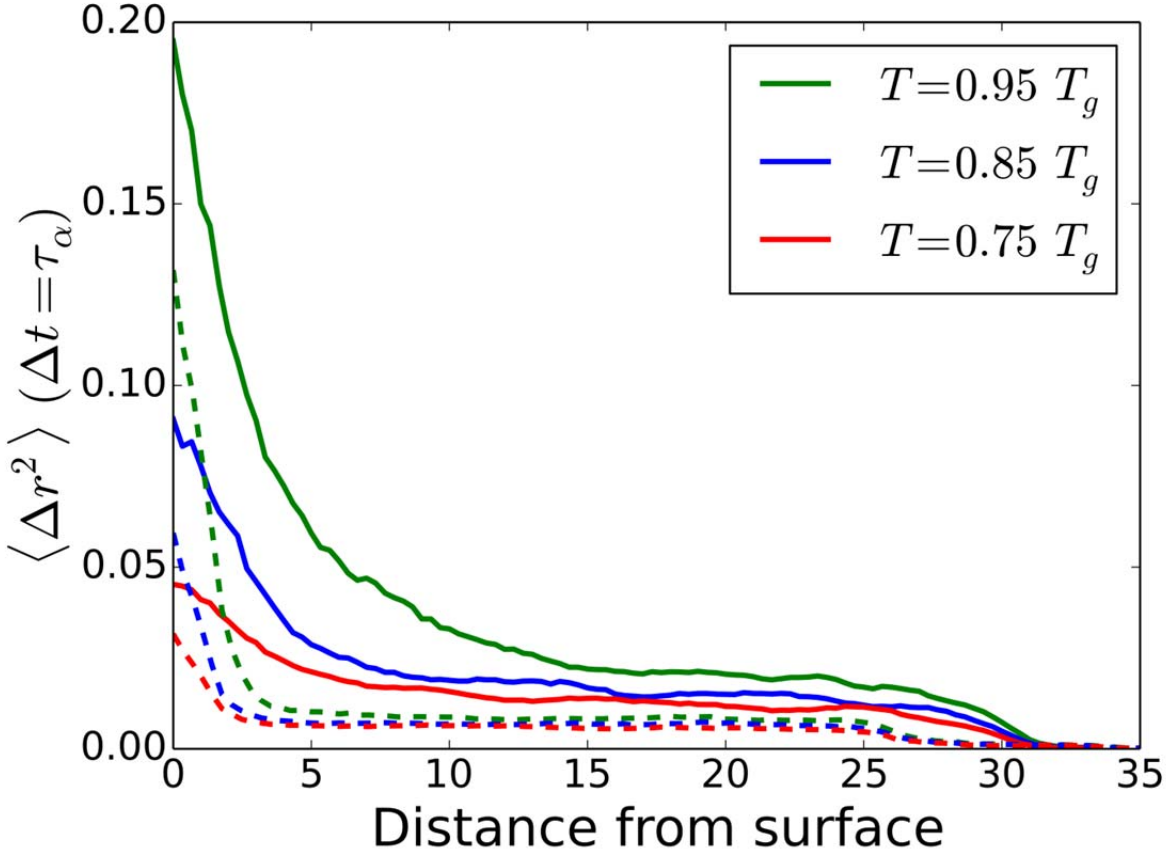

Figure 8: Mobility data for 2D and 3D PVD films equilibrated at several temperatures, all formed using NVE deposition at t_{dep} = 1.4X10^1 τ_α, which corresponds to roughly the same growth rate for both types of films when growth rates are normalized by τ_α. Dashed lines represent 3D films, while solid represent 2D films. The fraction of species A, χ_A = 0.65, 0.80 and T_g = 0.21, 0.335 in 2D and 3D, respectively. <r^2> is measured for $\Delta t = \tau_\alpha$. As temperature increases, the mobile layer extends further into the film. At the same multiple of T_g, the mobile layer in 2D always extends further. At this deposition rate, the growth rate of 2D and 3D films are nearly equal.

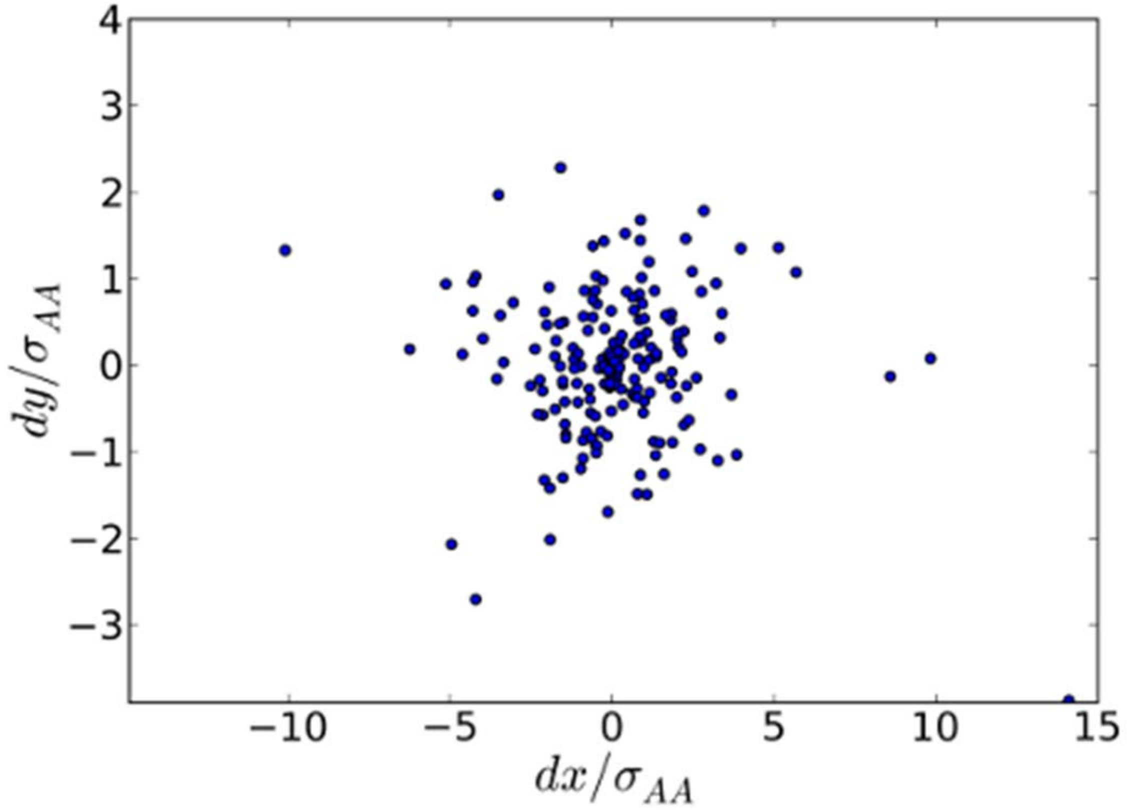

Figure 9: Change in x and y from vapor atoms' position at initial touchdown and cooling to their position once 300 more atoms have been deposited and the tracked atoms have been glassified. Values shown are for type A atoms only. Films are formed using $t_{dep} = 1.4X10^4\ \tau_{\alpha}$.

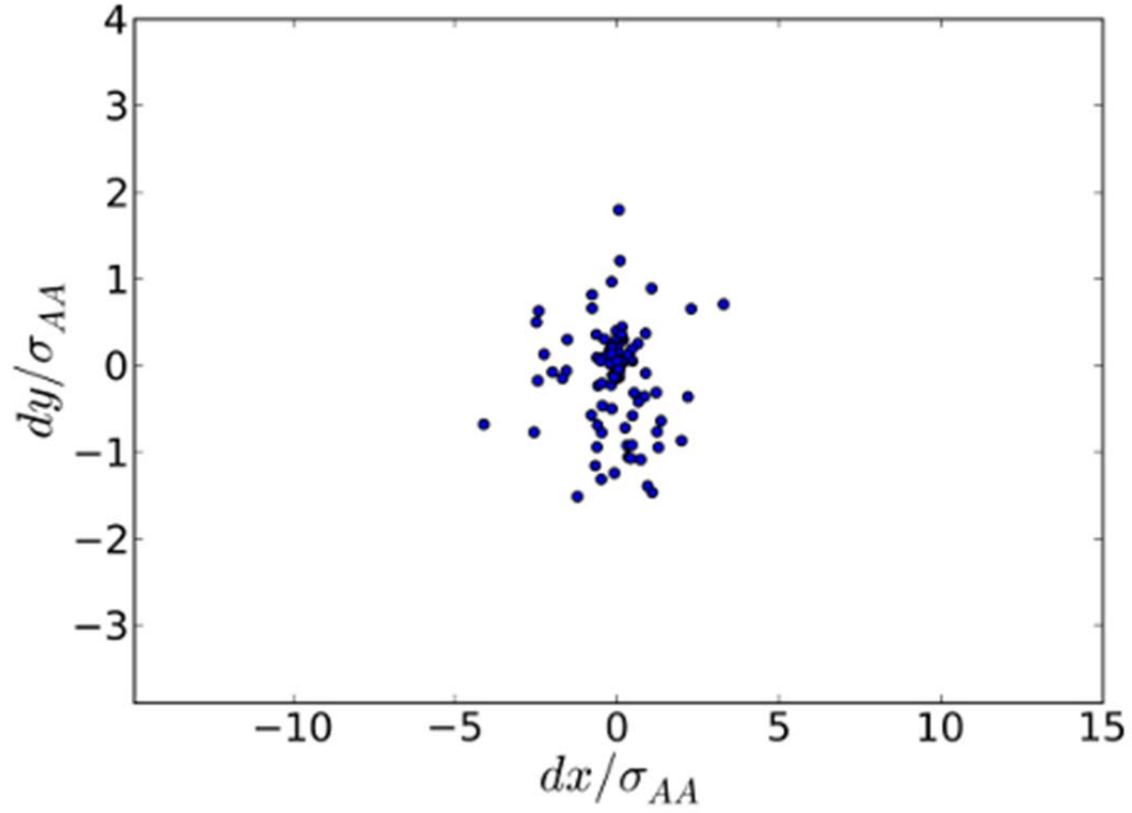

Figure 10: Change in x and y from vapor atoms' position at initial touchdown and cooling to their position once 300 more atoms have been deposited and the tracked atoms have been glassified. Values shown are for type B atoms only. Films are formed using t_{dep} = 1.4X10^4 τ_{α}.

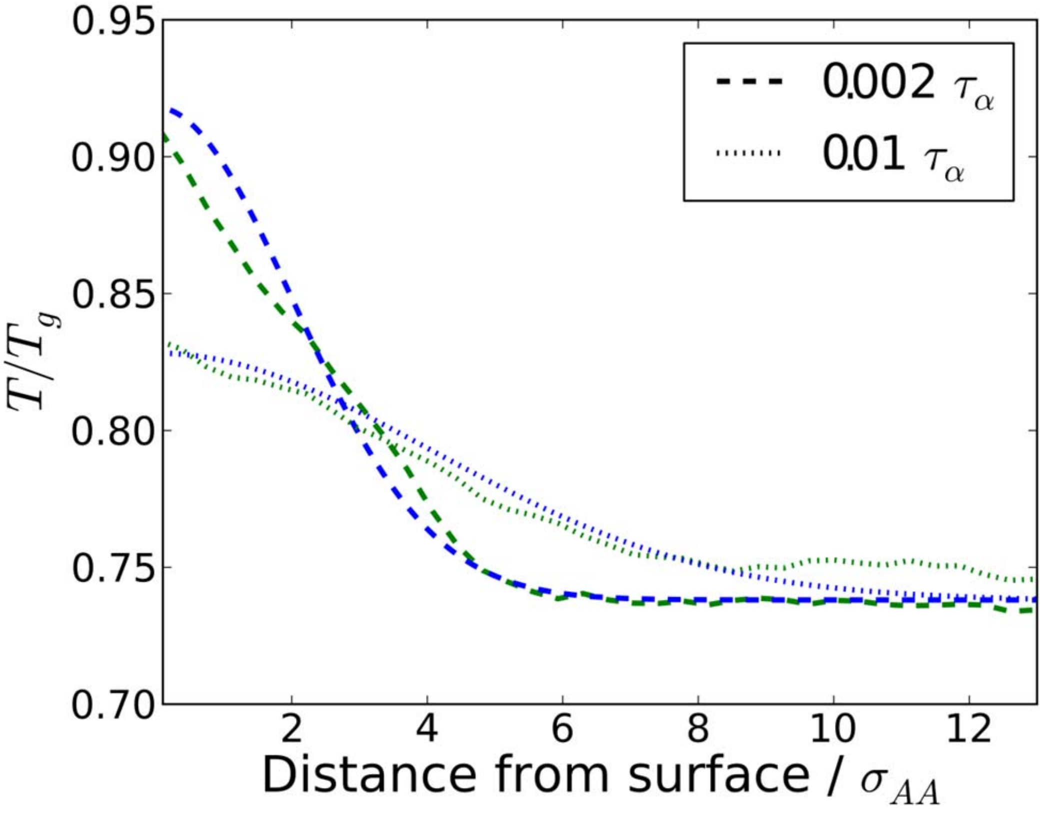

Figure 11: Temperature profile for PVD films shortly after the impact of many vapors, and a 1-dimensional model which mimics a PVD film where heat only transfers by diffusion. PVD film data are shown in green while data from the continuum model are shown in blue. In contrast to material presented in the main text, where the impact of only one atom was considered, here we consider the impact of many atoms. As a result, the continuum results are recovered.

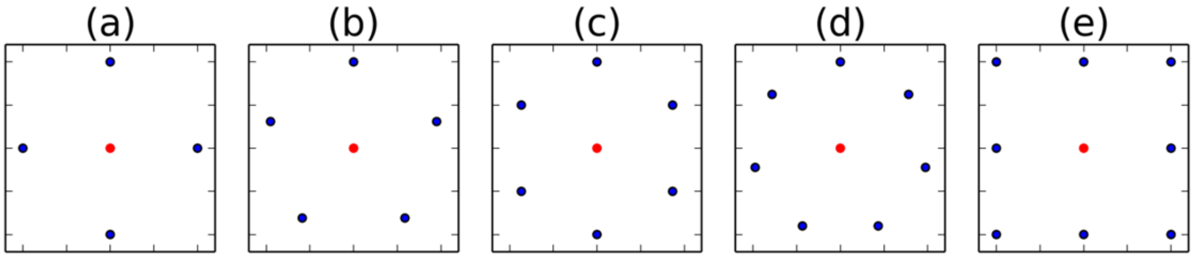

	(a)	(b)	(c)	(d)	(e)
q_5	0	0.701	0	0	0.415
q_8	64.0	0.273	3.79	0.273	64.0

Figure 12: Several sample configurations for which the q_5 and q_8 order parameters are calculated. The q parameters are calculated for the red center atoms of each configuration. Note that the q order parameters are based on spherical harmonics and thus depend only on the angle between the red center atom and each neighbor. Thus configurations such as an octagon and a square represented by 8 points as shown in (e) are equivalent. Also included are calculated q_5 and q_8 values for configurations (a) through (e). q_5 selects strongly for 5-fold symmetry (b) and weakly for 8-fold symmetry (e). The cutoff we use for high q_5 order is above the q_5 value for an 8-fold symmetric configuration as shown in (e). The q_8 parameter selected strongly for 4 and 8-fold symmetry, and very weakly for other symmetries.

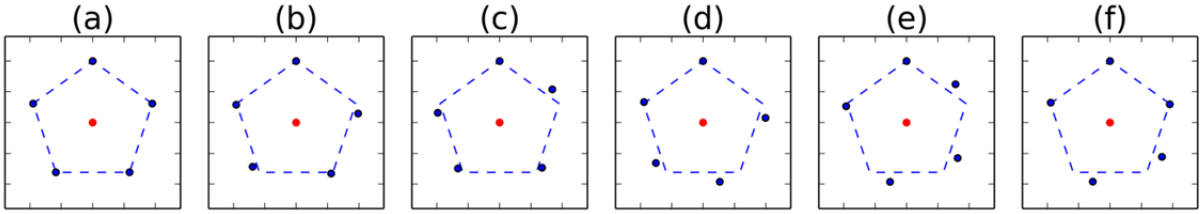

	(a)	(b)	(c)	(d)	(e)	(f)
q_5	0.71	0.65	0.60	0.55	0.50	0.45
q_8	0.00	23.4	44.7	16.7	21.5	16.5

Figure 13 Several sample configurations for which the q_5 and q_8 order parameters are calculated. The calculated values are shown in the associated table. The blue dashed line represents a perfect pentagon. The q parameters are calculated for the red center atoms of each configuration. The configurations range from a perfect pentagon (a) which yields a maximum q_5 value to configurations which yield lower q_5 values. While these configurations are representative, they are by no means the only configurations which yield these values. The configurations were generated by starting with a perfect pentagon and randomly rotating points around the center until the desired q_5 value was reached. Panel (d) represents a q_5 value of 0.55, that used as a cutoff in the main work.

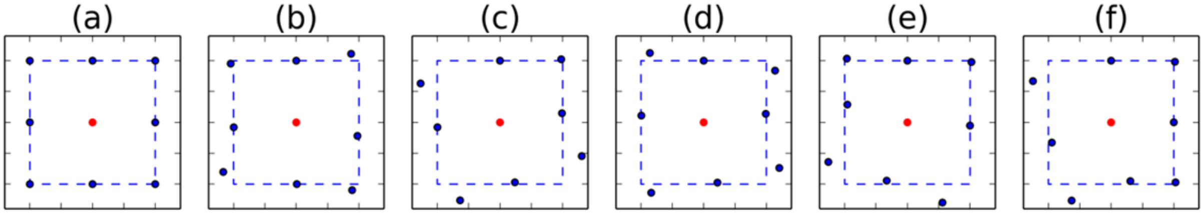

	(a)	(b)	(c)	(d)	(e)	(f)
q_5	0.00	0.22	0.09	0.16	0.14	0.14
q_8	64.0	40.0	30.0	24.0	22.0	20.0

Figure 14: Several sample configurations for which the q_5 and q_8 order parameters are calculated. The calculated values are shown in the associated table. The blue dashed line represents a perfect square, which yields a maximum q_8 value. The q parameters are calculated for the red center atoms of each configuration. The configurations range from a perfect square (a) which yields a maximum q_8 value to configurations which yield lower q_8 values. The configurations were generated by starting with a perfect square and randomly rotating points around the center until the desired q_8 value was reached. Panel (e) represents a q_8 value of 22, that used as a cutoff in the main work. Note that while many of these configurations appear similar to the eye, different cutoffs can distinguish between varying degrees of order. For example, increasing the high-order q_8 cutoff from that used for (e) to that used for (d) significantly decreases the fraction of high-order atoms, as can be seen comparing Figure 9 in the main work to Figures 17 and 20 in the Supplementary Information.

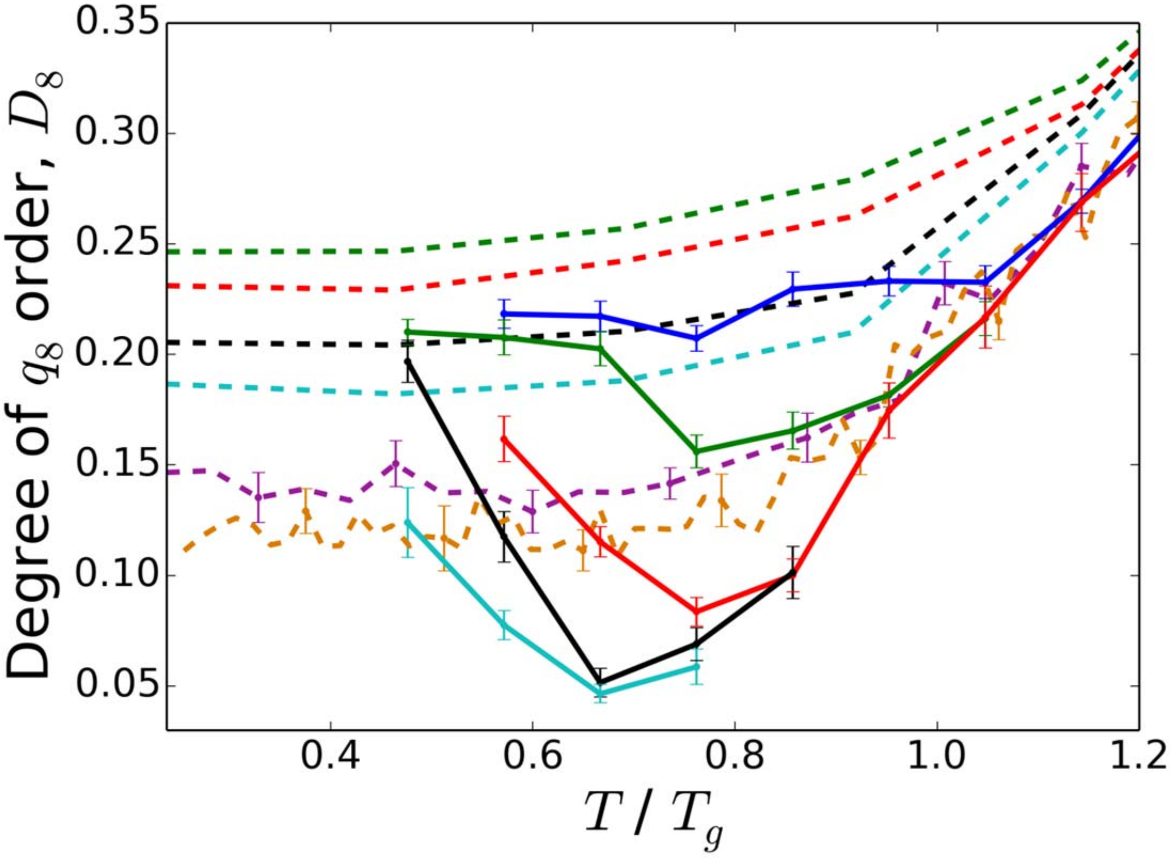

Figure 15: Fraction of highly q_8 ordered atoms for vapor deposited and liquid cooled films. Solid lines represent vapor deposited films, while dashed lines represent liquid cooled. The color of each line represents the rate at which its films were formed, as described in the main text. The data here was generated using a q_8 cutoff value of 24, rather than 22 as in the main text.

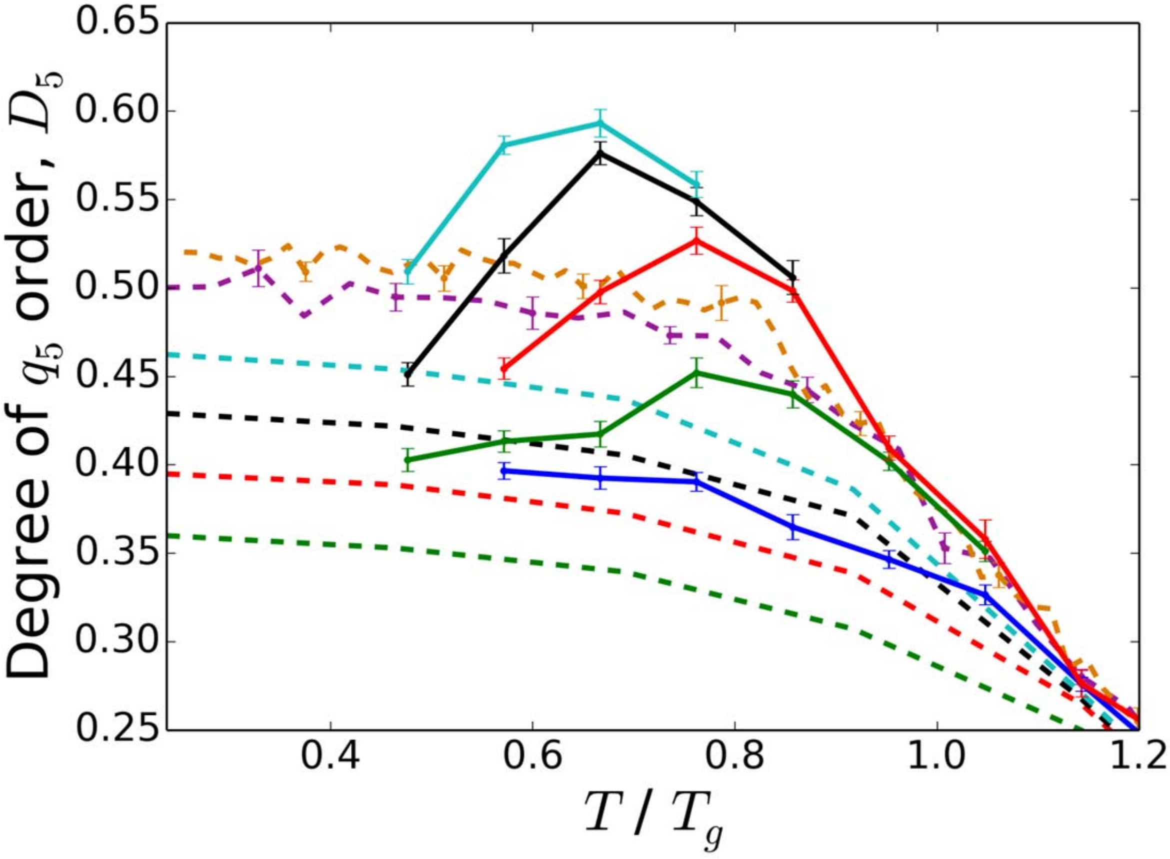

Figure 16: Fraction of highly q_5 ordered atoms for vapor deposited and liquid cooled films. Solid lines represent vapor deposited films, while dashed lines represent liquid cooled. The color of each line represents the rate at which its films were formed, as described in the main text. The data here was generated using a q_5 cutoff value of 0.60, rather than 0.55 as in the main text.

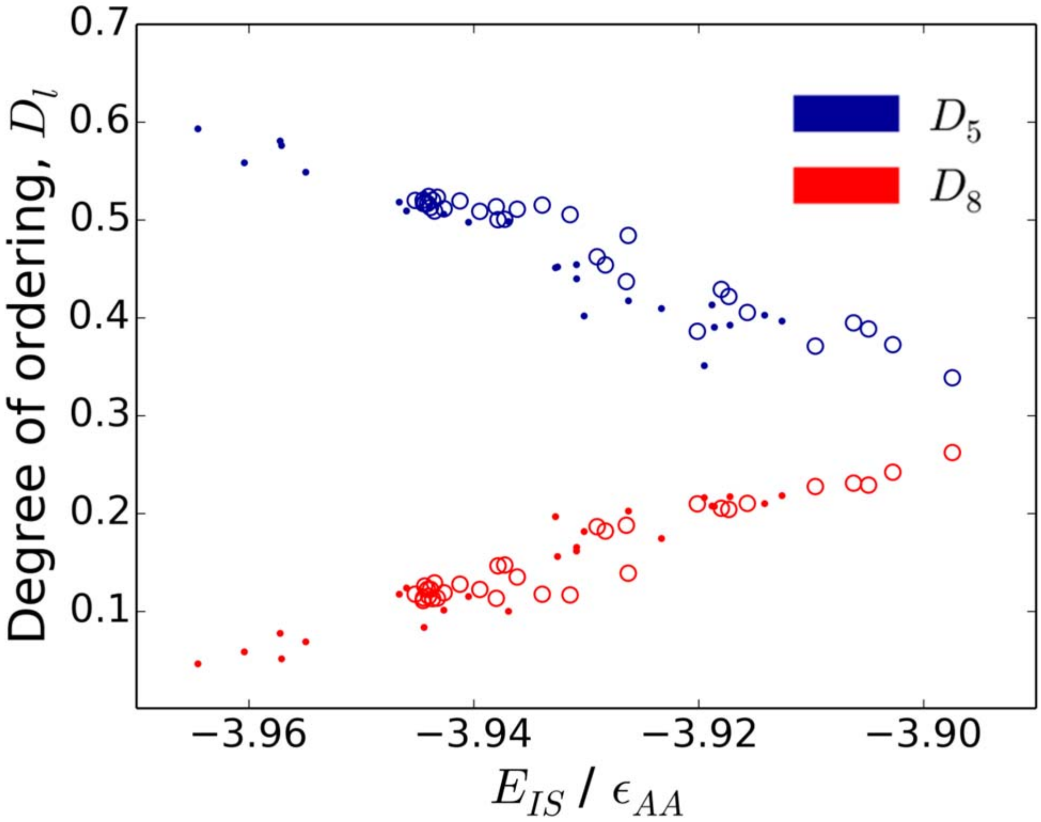

Figure 17: Degree of q_5 and q_8 ordering, D_l, for vapor deposited and liquid cooled thin films vs. inherent structural energy, E_{IS}. Solid circles represent vapor deposited data while open circles represent liquid cooled data. Films used are identical to those used in the main text. This data uses the high crystallinity thresholds as shown in Figures 15 and 16.

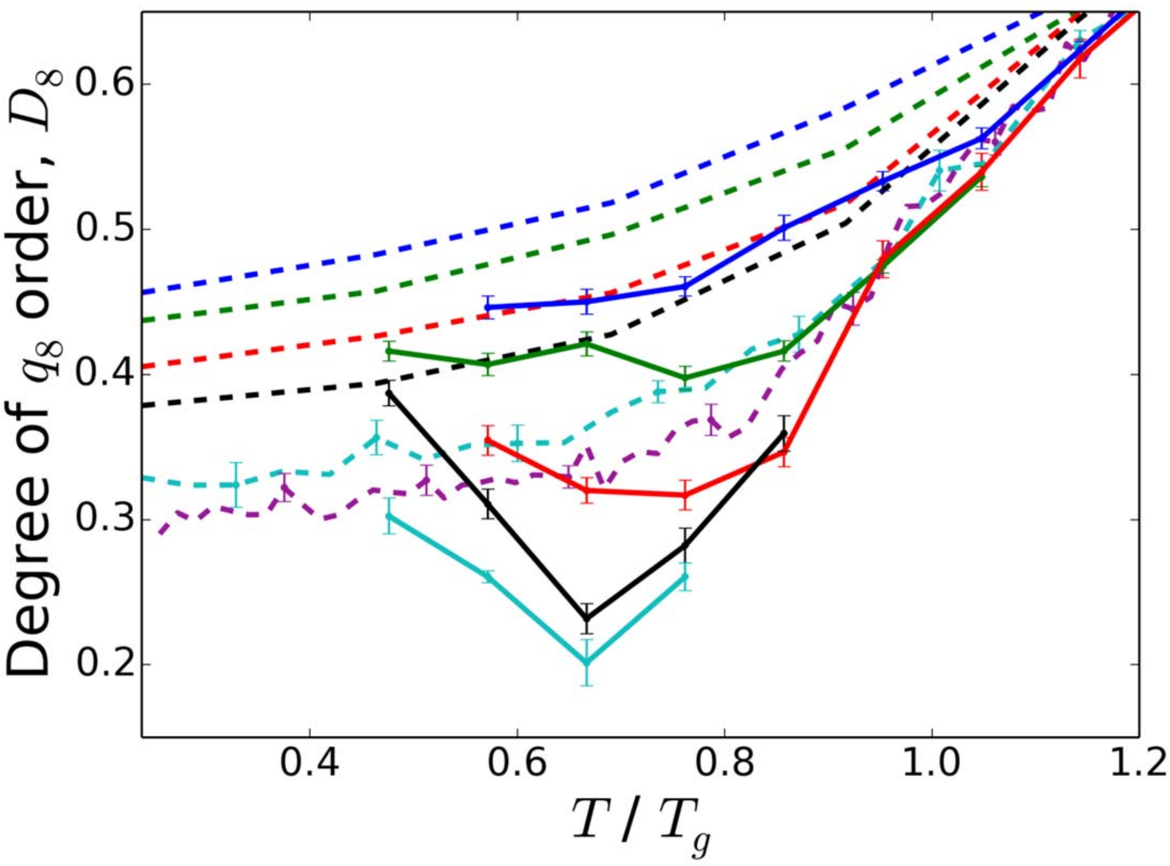

Figure 18: Fraction of highly q_8 ordered atoms for vapor deposited and liquid cooled films. Solid lines represent vapor deposited films, while dashed lines represent liquid cooled. The color of each line represents the rate at which its films were formed, as described in the main text. The data here was generated using a q_8 cutoff value of 20, rather than 22 as in the main text.

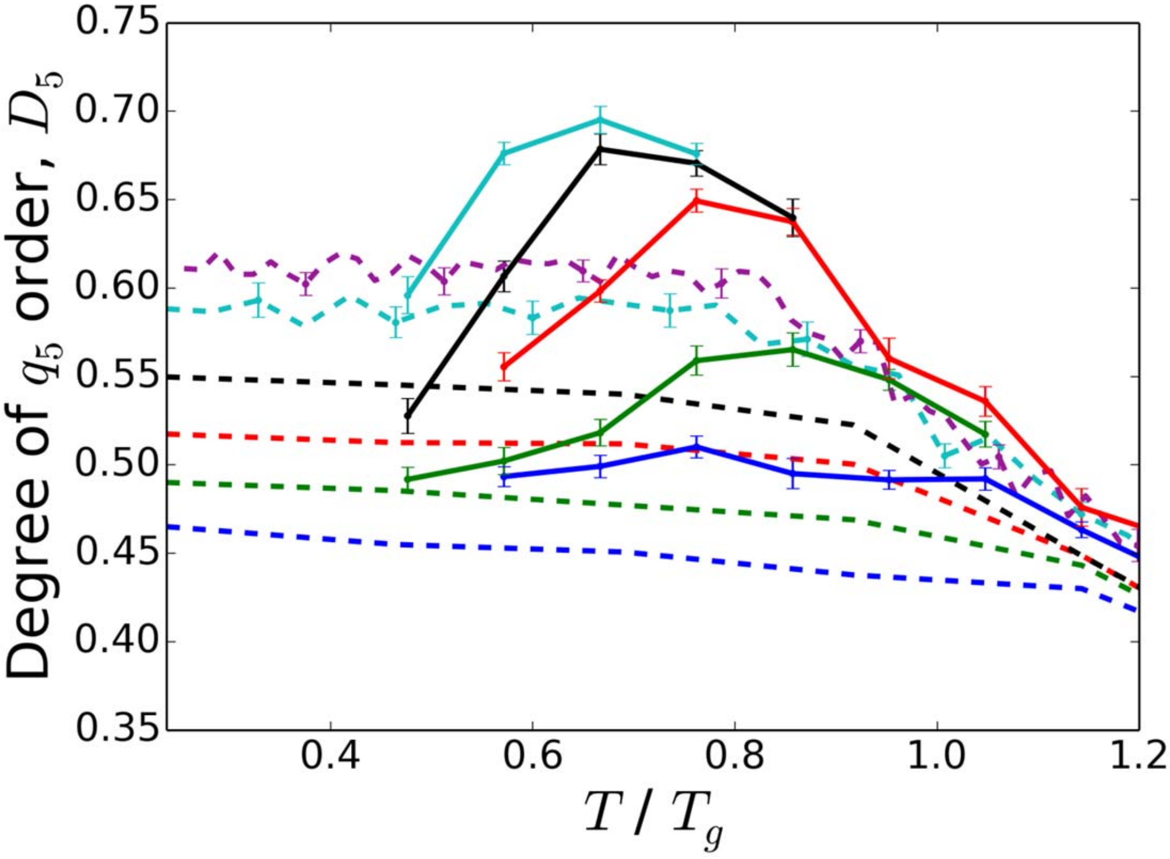

Figure 19: Fraction of highly q_5 ordered atoms for vapor deposited and liquid cooled films. Solid lines represent vapor deposited films, while dashed lines represent liquid cooled. The color of each line represents the rate at which its films were formed, as described in the main text. The data here was generated using a q_5 cutoff value of 0.50, rather than 0.55 as in the main text.

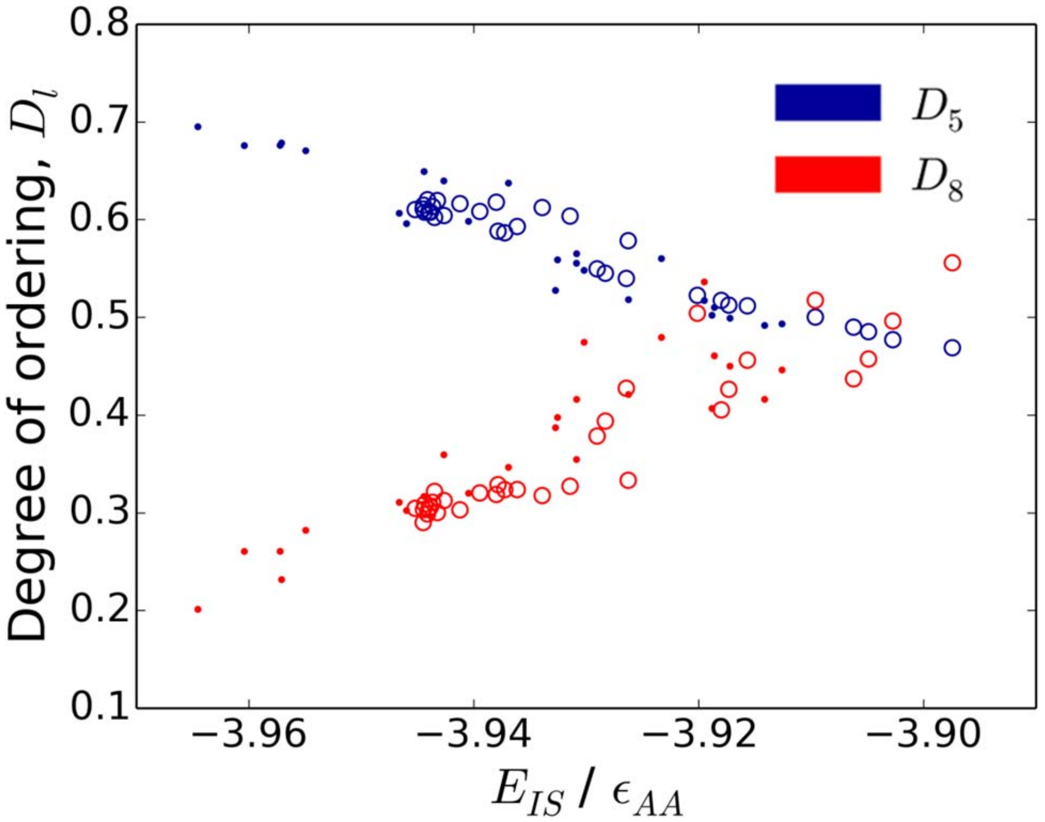

Figure 20: Degree of q_5 and q_8 ordering, D_l, for vapor deposited and liquid cooled thin films vs. inherent structural energy, E_{IS}. Solid circles represent vapor deposited data while open circles represent liquid cooled data. Films used are identical to those used in the main text. This data uses the lower crystallinity thresholds as shown in Figures 18 and 19.

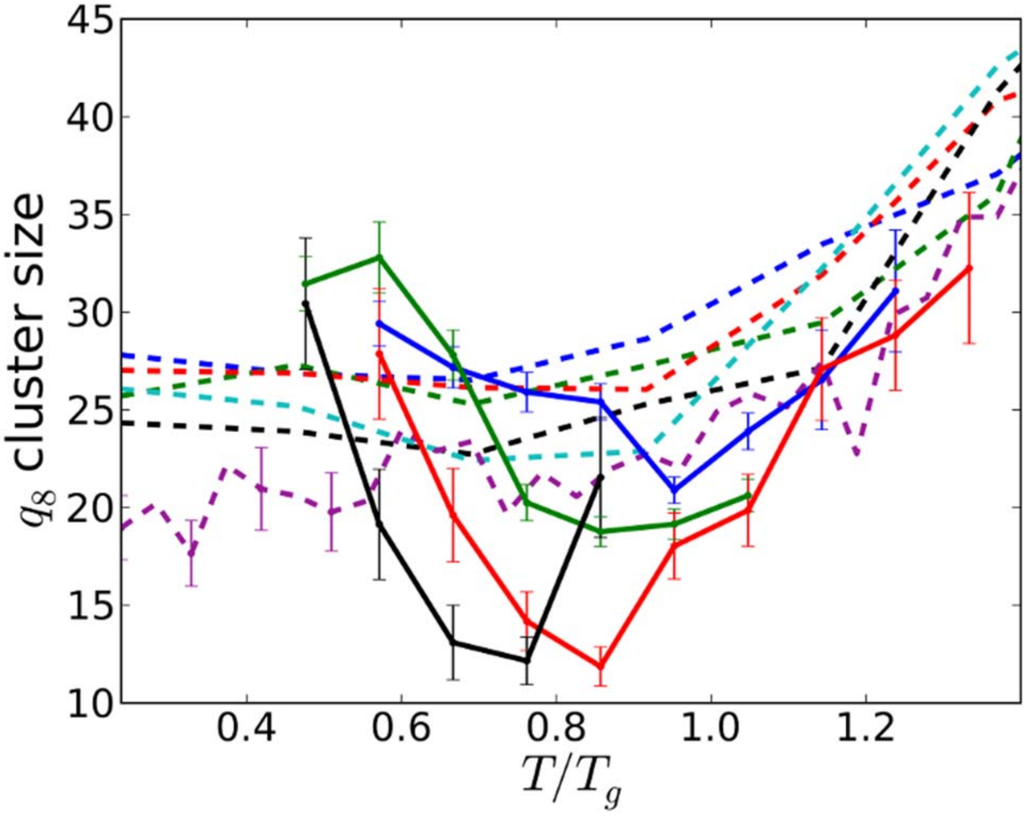

Figure 21: Average size of q_8 high-order clusters for liquid cooled and vapor deposited films as discussed in the main text. Cluster size is reported in number of atoms. Dashed lines represent liquid-cooled data while solid lines represent vapor deposited data. We find that ordered q_8 domains grow smaller with film stability and decreasing D_8. The noise in this data is due to the low number of q_8 clusters found in each sample. The data shows that the q_8 cluster sizes for vapor deposited and liquid-cooled films are roughly equivalent for films of equal energy. Error bars represents 95% confidence intervals. As films become very stable, they show so few q_8 clusters that the data become noisy. Thus, cluster size data for our most stable films is not included.

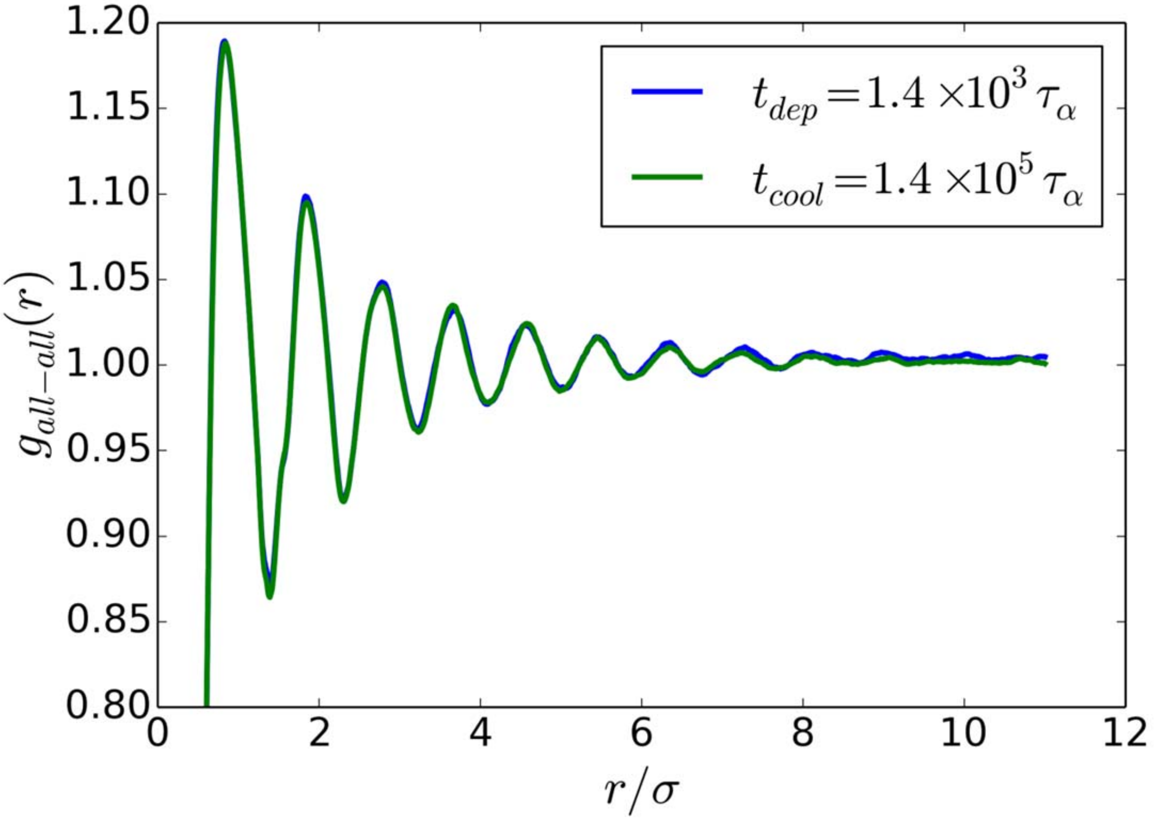

Figure 22: Radial distribution function for all-all in liquid cooled films formed with t_{cool} = 1.4X10^5 τ_α at T=0.16 and PVD films formed with t_{dep}=1.4X10^3 τ_α with T_s = 0.16.

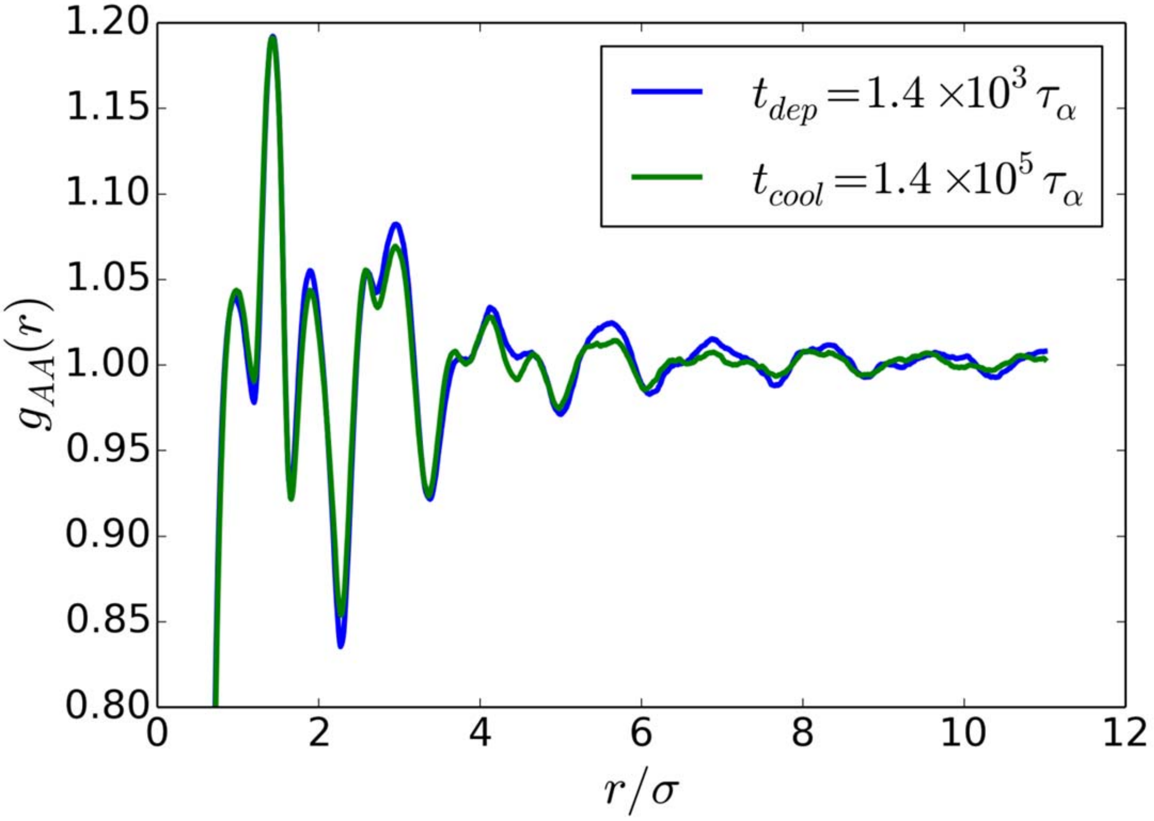

Figure 23: Radial distribution function for A-A in liquid cooled films formed with t_{cool} = 1.4X10^5 τ_α at T=0.16 and PVD films formed with t_{dep}=1.4X10^3 τ_α with T_s = 0.16.

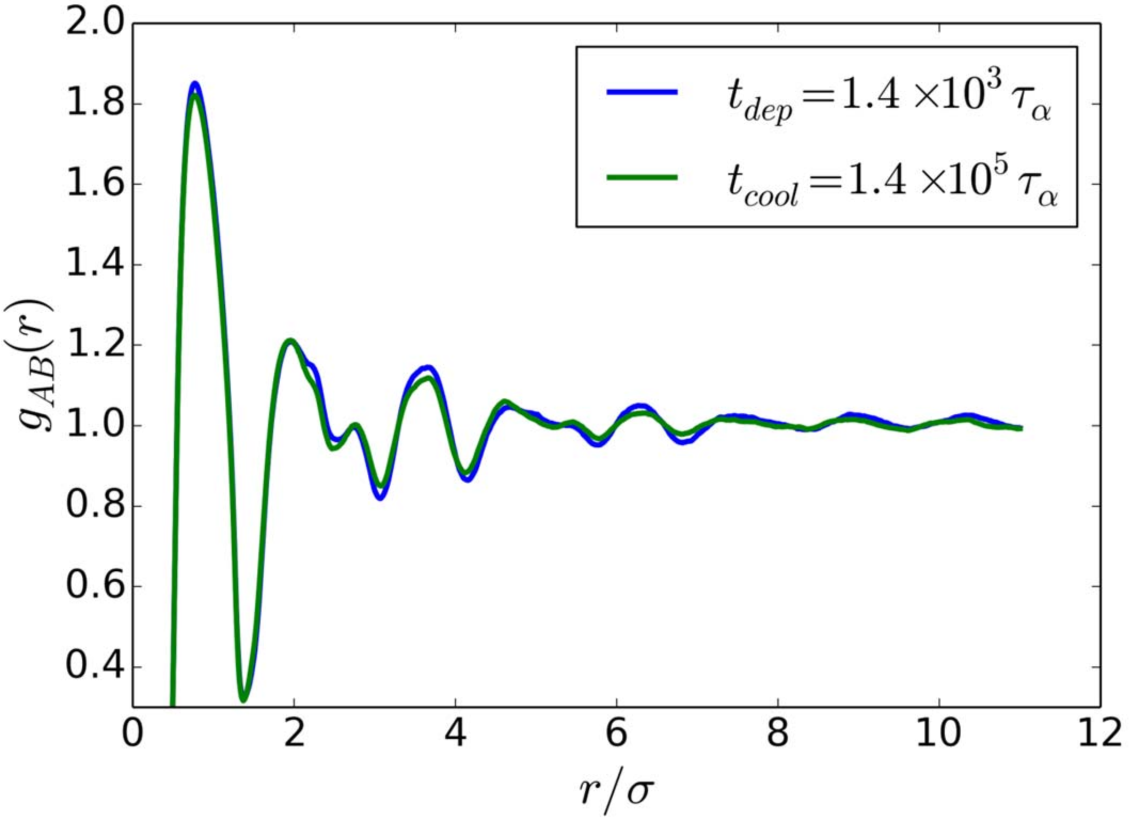

Figure 24: Radial distribution function for A-B in liquid cooled films formed with t_{cool} = 1.4X10^5 τ_α at T=0.16 and PVD films formed with t_{dep}=1.4X10^3 τ_α with T_s = 0.16.

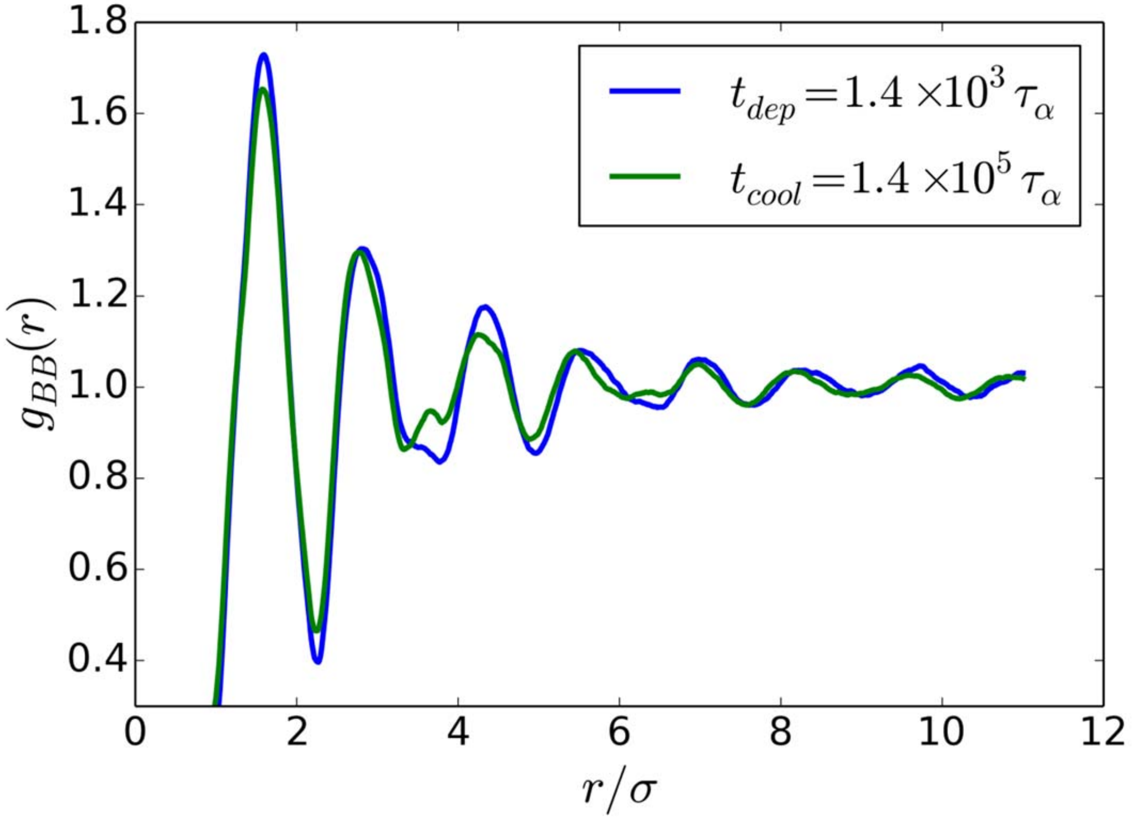

Figure 25: Radial distribution function for B-B in liquid cooled films formed with t_{cool} = 1.4X10^5 τ_α at T=0.16 and PVD films formed with t_{dep}=1.4X10^3 τ_α with T_s = 0.16.

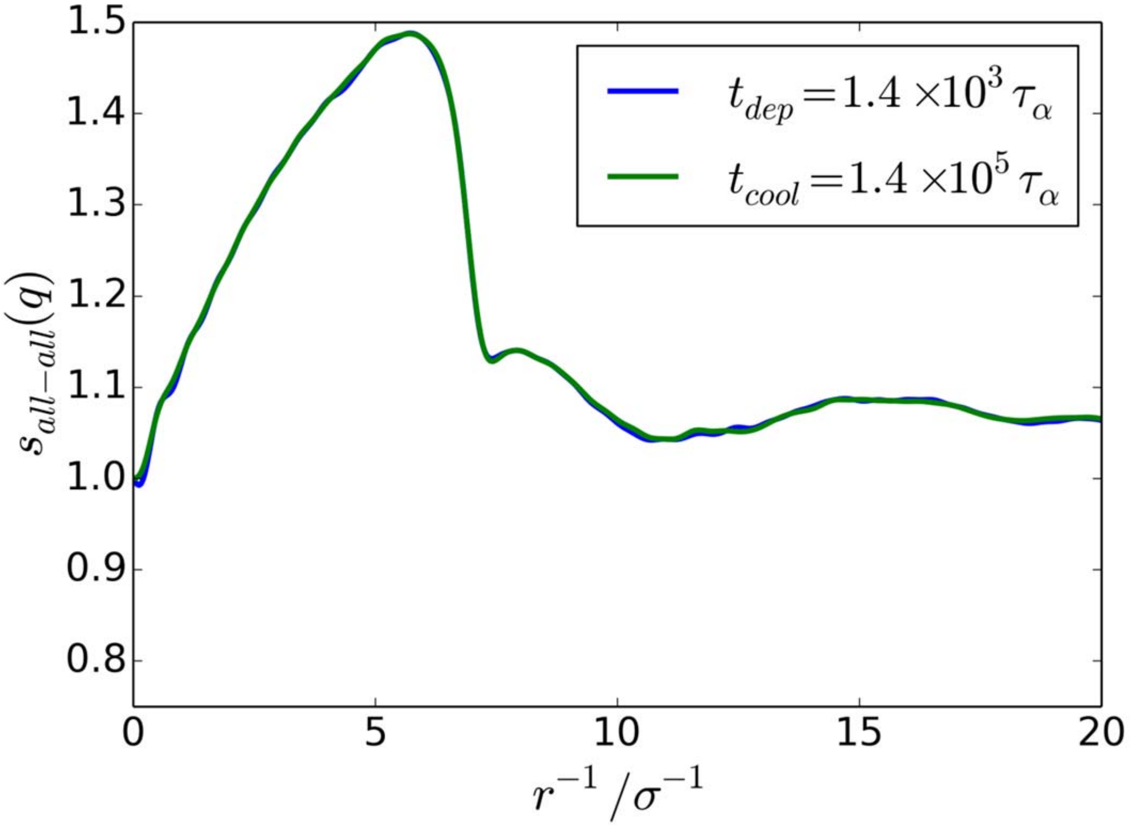

Figure 26: Structure factor for all-all in liquid cooled films formed with t_{cool} = 1.4X10^5 τ_α at T=0.16 and PVD films formed with t_{dep}=1.4X10^3 τ_α with T_s = 0.16.

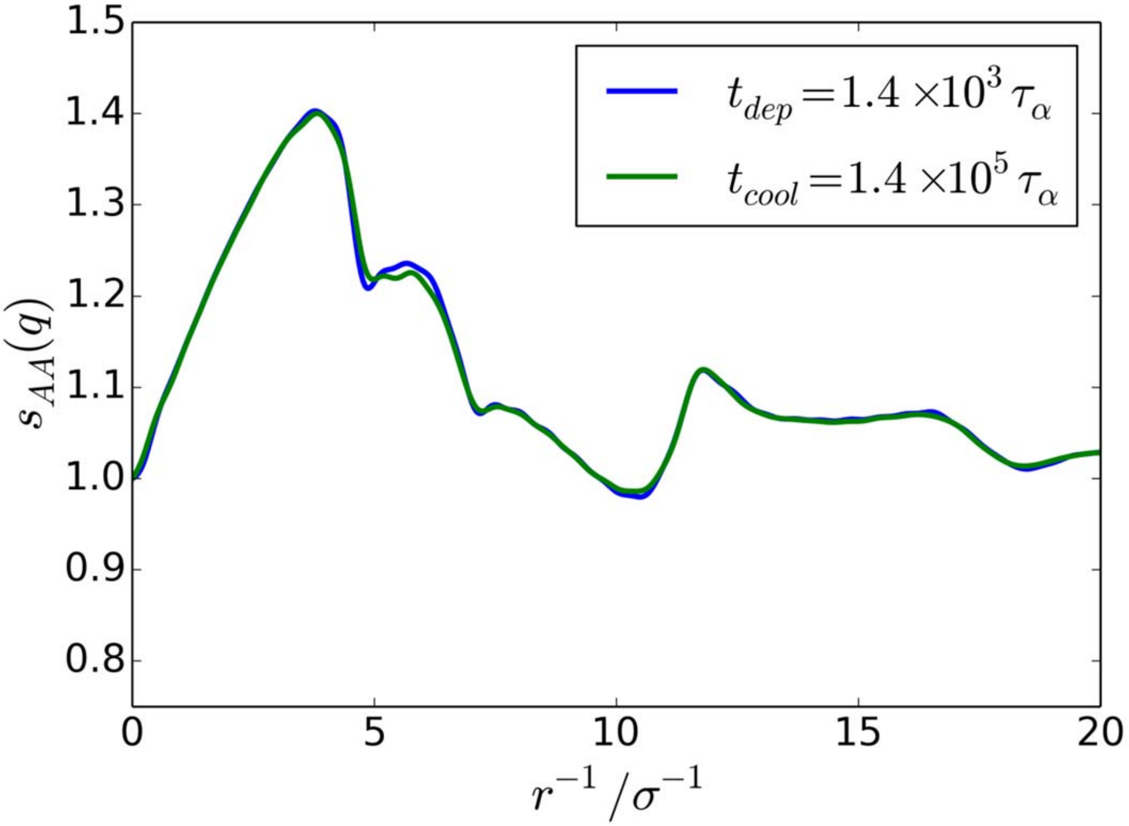

Figure 27: Structure factor for A-A in liquid cooled films formed with t_{cool} = 1.4X10^5 τ_α at T=0.16 and PVD films formed with t_{dep}=1.4X10^3 τ_α with T_s = 0.16.

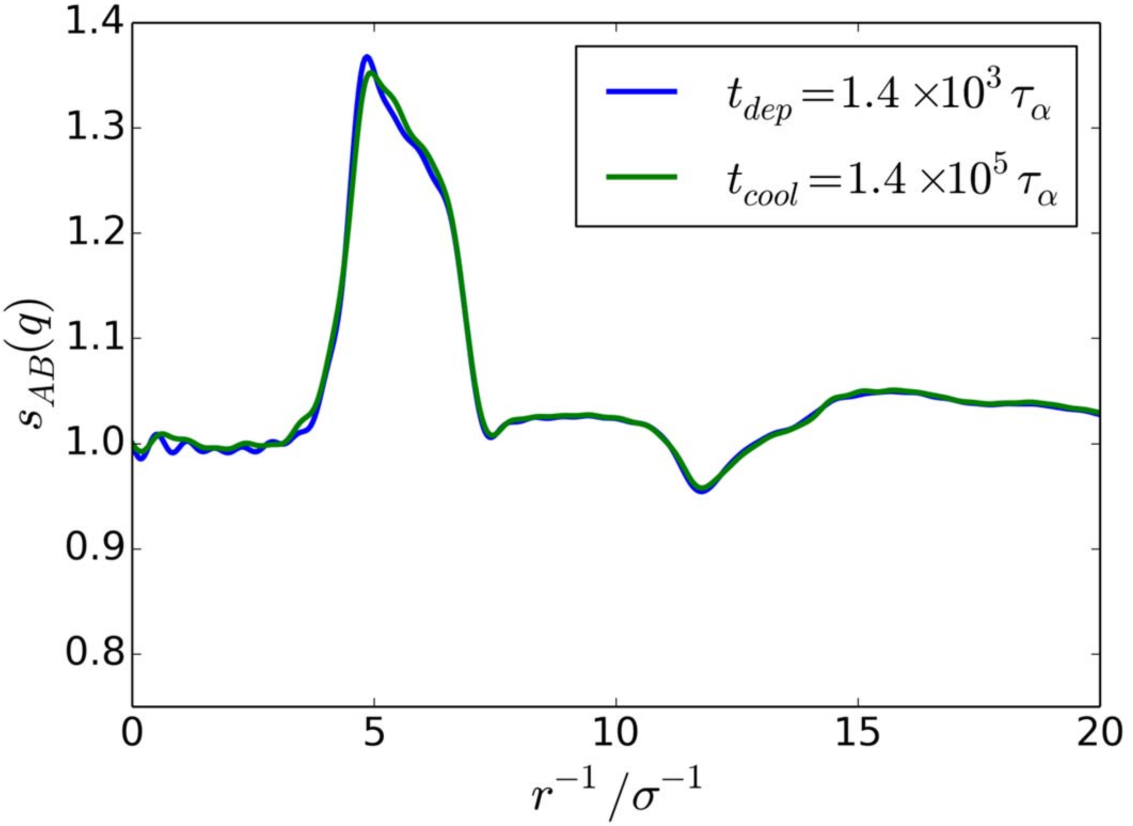

Figure 28: Structure factor for A-B in liquid cooled films formed with $t_{cool} = 1.4X10^5\, \tau_\alpha$ at T=0.16 and PVD films formed with t_{dep}=$1.4X10^3\, \tau_\alpha$ with T_s = 0.16.

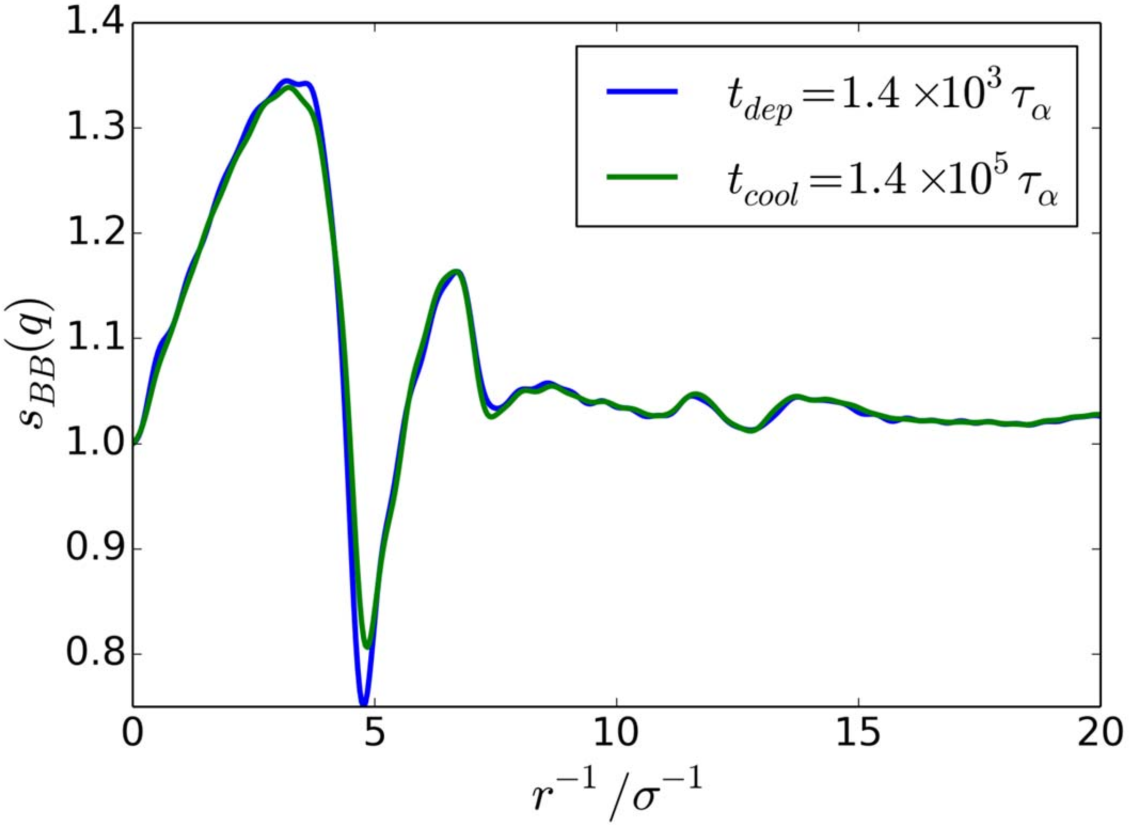

Figure 29: Structure factor for B-B in liquid cooled films formed with t_{cool} = $1.4X10^5\,\tau_\alpha$ at T=0.16 and PVD films formed with t_{dep}=$1.4X10^3\,\tau_\alpha$ with T_s = 0.16.

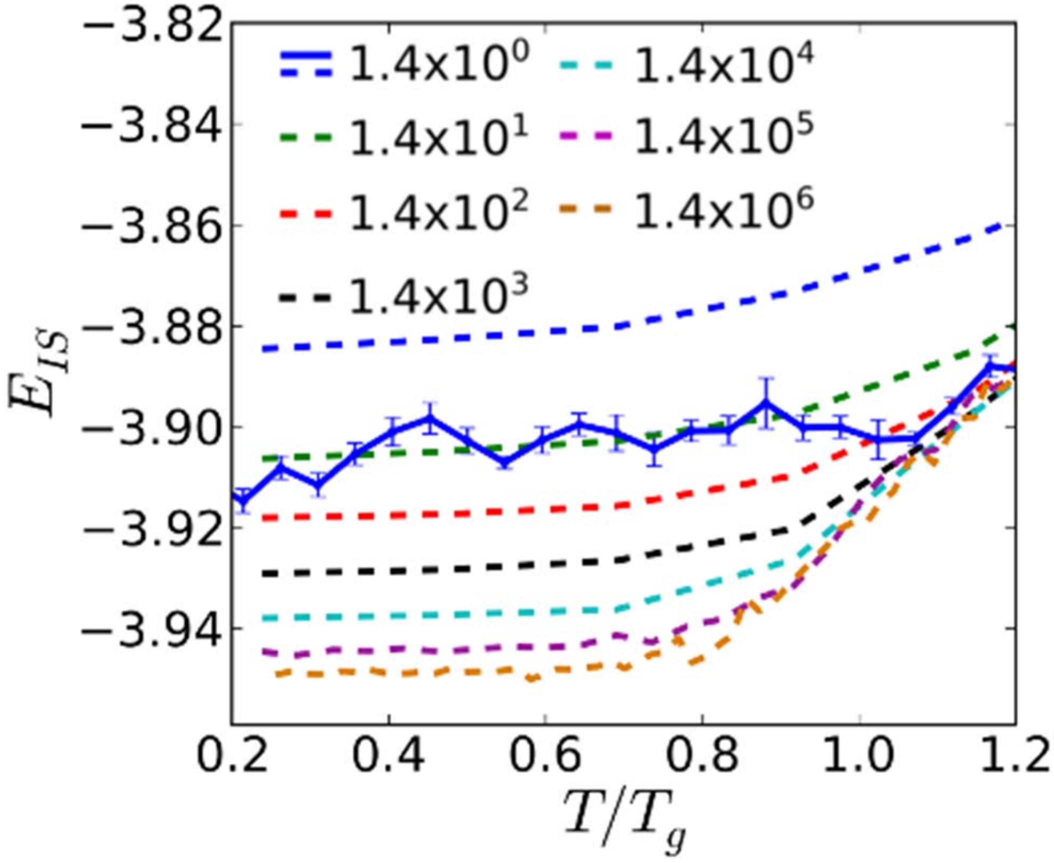

Figure 30: Inherent structural energies for liquid cooled and NVT deposited 2D films. Dotted lines represent liquid cooled energies and solid lines represent vapor deposited. All conditions in these simulations are identical to those done in the main work except for the deposition style. Films produced by NVT deposition are significantly less stable than those produced by NVE deposition. NVT deposition in 2D also fails to show an ideal substrate temperature as is shown in both experiment and NVE deposition. Previous work has employed thermostats to control the temperature of the substrate, film, and newly added atoms, with new atoms being cooled linearly to the film temperature (NVT deposition). In this work, a thermostat is only applied to the substrate, which acts as a heat bath for the rest of the film, which has no thermostat applied to it (NVE deposition).

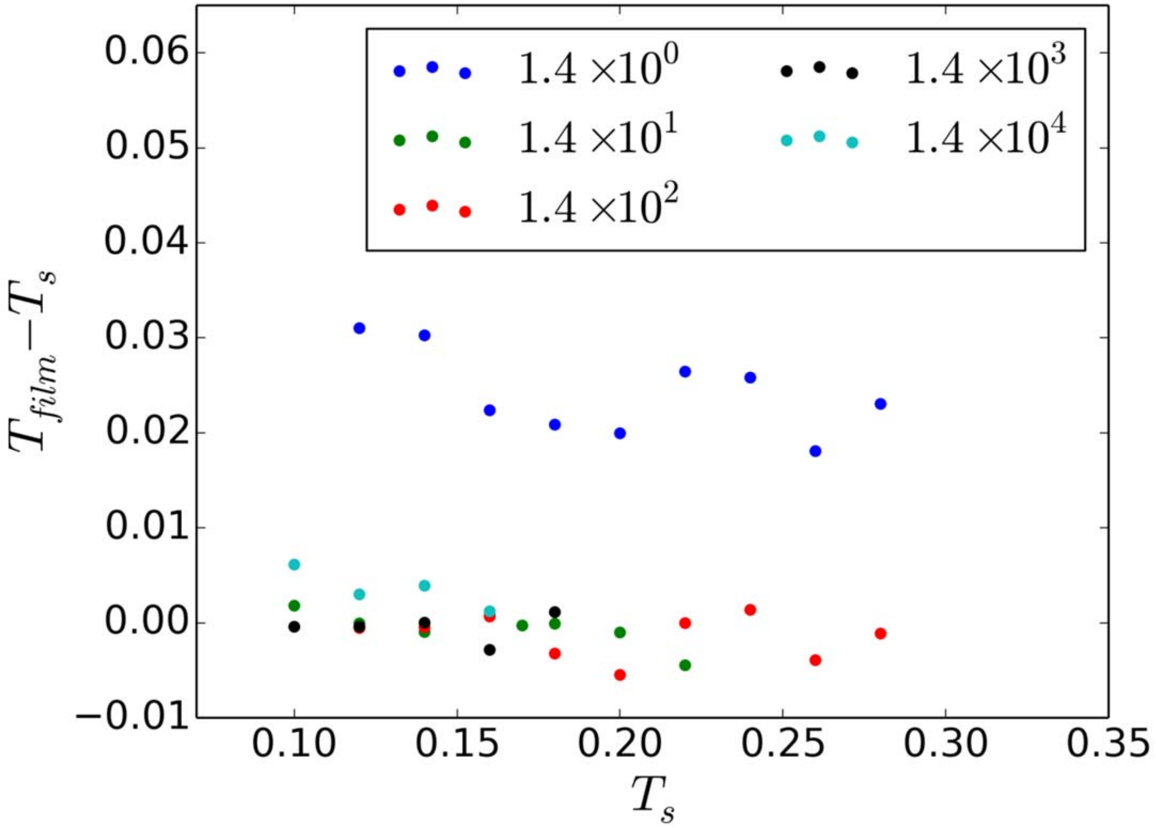

Figure 31: Deviation of film temperatures from substrate temperatures for all deposition rates and substrate temperatures. Legend values refer to t_{dep} in units of τ_α measured at T=1.10 T_g. The deviation occurs due to the heat from vapor atoms. Heat is absorbed by the substrate, which is held under at constant temperature. If deposition occurs quickly, a temperature gradient through the film occurs. Each point represents the average of the instantaneous temperature of ten 35 σ thick films. At equilibrium, the temperatures of the film and substrate will be equal to within thermal fluctuations. However if vapor atoms are added too rapidly, the film temperature could deviate from that of the substrate. For films deposited with t_{dep}=1.4X10^0 τ_α, T_{film} was used for all calculations. For all other deposition rates, T_s was used in calculations.

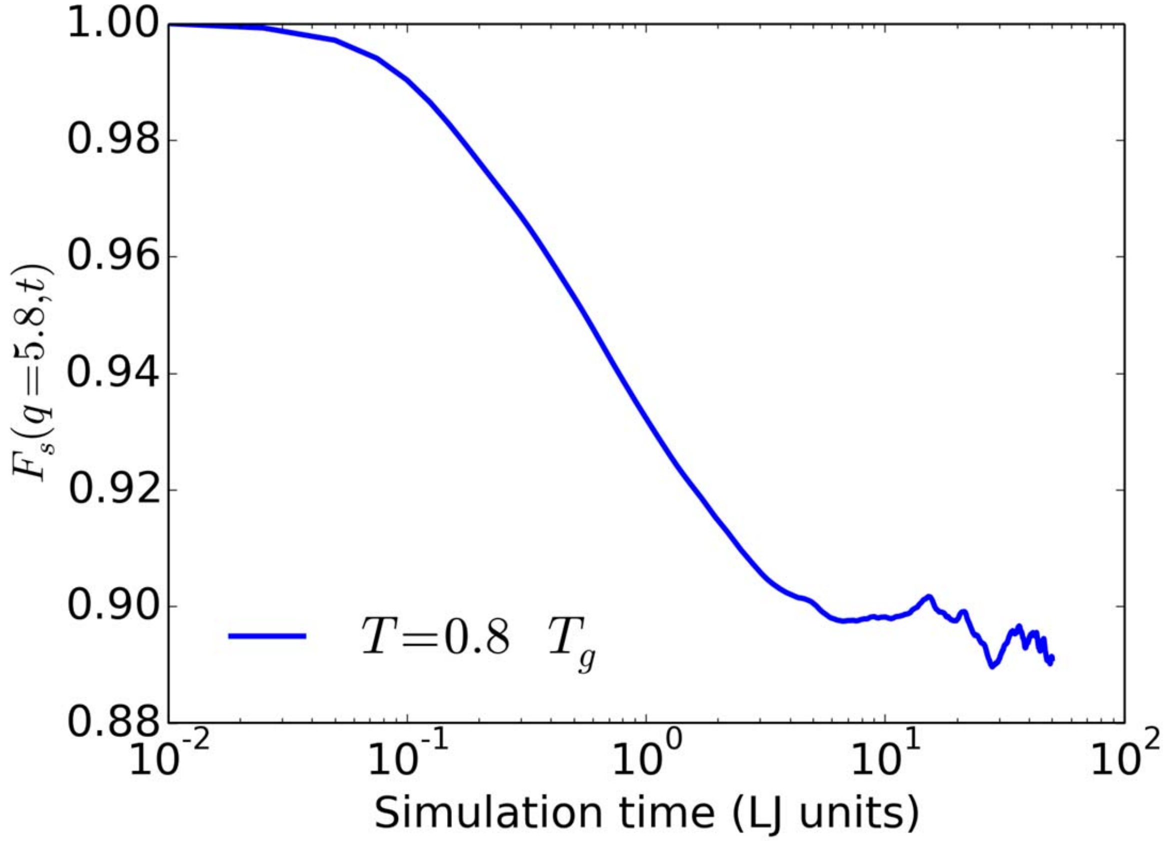

Figure 32: Self-intermediate scattering function for 2D films at T = 0.8 T_g calculated with q=5.8, as determined from the static structure factor. We use data from this figure to estimate the in-cage relaxation time, τ_β, which is used to time-average positions for analysis of local order. The time must be at least long enough for particles to sample configurations within the local cage. For this reason, we use the time where the scattering function has fully decayed to its plateau value, 10 Lennard-Jones time units.

Supplementary Table 1

t_{dep} / τ_α	T_S/T_g	T_{cool}/ τ_α
1.4X10^0	0.87	1.5X10^2
1.4X10^1	0.79	4.0X10^3
1.4X10^2	0.73	1.4X10^5
1.4X10^3	0.70	4.7X10^7
1.4X10^4	0.68	1.6X10^{10}

Table 1: Predicted liquid cooling time required to form films with energy equal to vapor deposited samples deposited with ideal substrate temperature. Predicted liquid cooling rates are calculated using Equation 1 in the main text. Ideal substrate temperatures are found by fitting a cubic spline to E_{IS} vs. T_s at a given t_{dep} using data shown in Figure 3 in the main text.
